# Return to sports after COVID-19: a position paper from the Dutch Sports Cardiology Section of the Netherlands Society of Cardiology

**DOI:** 10.1007/s12471-020-01469-z

**Published:** 2020-07-13

**Authors:** G. C. Verwoert, S. T. de Vries, N. Bijsterveld, A. R. Willems, R. vd Borgh, J. K. Jongman, H. M. C. Kemps, J. A. Snoek, R. Rienks, H. T. Jorstad

**Affiliations:** 1 Department of Cardiology, Amsterdam University Medical Centre, Amsterdam, The Netherlands; 2 Department of Cardiology, Erasmus Medical Centre, Rotterdam, The Netherlands; 3 Department of Cardiology, Tjongerschans Hospital, Heerenveen, The Netherlands; 4 Department of Cardiology, Flevo Hospital, Almere, The Netherlands; 5 Department of Cardiology, OLVG, Amsterdam, The Netherlands; 6 Department of Cardiology, Laurentius Hospital, Roermond, The Netherlands; 7 Department of Cardiology, Wilhelmina Hospital, Assen, The Netherlands; 8 Department of Cardiology, Maxima Medical Centre, Veldhoven, The Netherlands; 9 Department of Industrial Design, Technical University, Eindhoven, The Netherlands; 10 Department of Sports medicine, Isala, Zwolle, The Netherlands; 11 Isala Heart Centre, Zwolle, The Netherlands; 12 Central Military Hospital, Utrecht, The Netherlands

**Keywords:** COVID-19, Sports, Exercise, Myocarditis, Recommendations

## Abstract

The coronavirus disease 2019 (COVID-19) pandemic has led to preventive measures worldwide. With the decline of infection rates, less stringent restrictions for sports and exercise are being implemented. COVID-19 is associated with significant cardiovascular complications; however there are limited data on cardiovascular complications and long-term outcomes in both competitive (elite) athletes and highly active individuals. Based on different categories of disease severity (asymptomatic, regional/systemic symptoms, hospitalisation, myocardial damage, and/or myocarditis), in this point-of-view article we offer the (sports) cardiologist or sports physician in the Netherlands a practical guide to pre-participation screening, and diagnostic and management strategies in all athletes >16 years of age after COVID-19 infection.

## Introduction

With the implementation of less stringent coronavirus disease 2019 (COVID-19) restrictions for sports and exercise, healthcare professionals are faced with an increasing number of athletes—both competitive (elite) athletes and highly active individuals (>3 h/week of exercise)—seeking return-to-sports advice after recovery from COVID-19. This is challenging, as evidence-based recommendations for a return to sports after infectious episodes are limited and heterogeneous. Different proposals regarding the resumption of sports and exercise after COVID-19, e.g. from the American College of Cardiology’s Sports and Exercise Cardiology Council, have recently been published [1, 2]. Based on different categories of disease severity, in this point-of-view article we offer the (sports) cardiologist or sports physician in the Netherlands a practical guide to pre-participation screening (PPS), and clinical management strategies in competitive (elite) athletes and highly active individuals >16 years of age after COVID-19.

## General recommendations

Healthcare professionals should stay up-to-date and adhere to the most recent national recommendations from the National Institute for Public Health and Environment [3]. Healthcare professionals directly involved in athlete care should also stay up to date with recommendations from the NOC*NSF [4] and other relevant sports organisations. If national recommendations do not cover specific circumstances, public health considerations should always be taken into account and no unnecessary risks are acceptable that could lead to further spread of severe acute respiratory syndrome coronavirus 2 (SARS-CoV-2).

## Cardiac considerations for COVID-19

Studies on critically ill, hospitalised patients have shown that infection with SARS-CoV‑2 is associated with several cardiovascular complications [5–8]. The mechanism of cardiac injury from COVID-19 has not yet been fully elucidated, but is likely multifactorial [9, 10]. For an overview of all described complications, see Tab. 1. The background of the cardiovascular complications is extensively discussed elsewhere in this issue of the *Netherlands Heart Journal*.

**Table 1:** Cardiac considerations in COVID-19

Mechanism of cardiovascular injury
Hypoxaemia
Myocardial infarction
Systemic inflammatory response syndrome/cytokine storms
Microvascular ischaemia
Myocarditis
Hypercoagulability

As the precise mechanisms and long-term consequences of the interaction between SARS-CoV‑2 and different (in particular cardiac) tissues are unknown, it is consequently unknown whether patients with (regional/systemic) symptoms are prone to cardiovascular sequelae after COVID-19 and what the influence of sports and exercise is on these potential sequelae.

## Specific considerations for athletes after COVID-19

Some considerations should be taken into account when assessing the scope of COVID-19 and potential cardiovascular complications in athletes. First, based on currently available reports, myocarditis leading to congestive heart failure and arrhythmias is diagnosed infrequently in patients with COVID-19, and no cases have been published describing elite athletes with SARS-CoV‑2 myocarditis [10–14]. However, all-cause myocarditis is a significant cause of sudden cardiac death and sudden cardiac arrest (SCD/SCA) in young athletes [15], with case series reporting myocarditis as a potential cause of SCD/SCA in up to 8% [16]. Second, athletes are not a high-risk group for a severe course of COVID-19. Athletes in general and elite athletes in particular are younger, which is associated with a milder course of COVID-19 [17]. They also have fewer cardiac comorbidities, and a lower prevalence of obesity, diabetes mellitus and hypertension. Third, moderate exercise training reduces the risk, duration, and severity of viral infections in general, and regular exercise has positive effects on pulmonary function [18].

However, severe cases of COVID-19 requiring hospitalisation have been described in younger individuals without comorbidities [19]. Furthermore, older athletes or athletes with cardiovascular comorbidities who are at risk for a severe course of COVID-19 will wish to resume sports and exercise. The optimal approach to sports resumption in these individuals is unclear, as data are lacking on cardiovascular complications and cardiac injury in patients with mild or asymptomatic COVID-19. Considering the possible severe consequences of cardiac complications of COVID-19 in athletes, we recommend that an early diagnostic evaluation should take place in individuals aiming to return to sports after recovery from COVID-19.

## Active COVID-19

Athletes with active COVID-19 should cease all sports activities and undergo self-isolation at home for the entire symptomatic period [4]. While COVID-19-specific data are lacking, waiting 7–14 days after resolution of all symptoms, and a minimum of 10 days after onset of symptoms before resuming exercise seems reasonable. After that, gradual resumption of exercise and sports can be considered, based on the severity and duration of symptoms and after stratification if PPS is indicated.

## After recovery from COVID-19

We stratify athletes that have recovered from the initial SARS-CoV‑2 infection as:

- asymptomatic or local symptoms (non-hospitalised)
- regional or systemic symptoms (non-hospitalised)
- hospitalised and no myocardial injury
- hospitalised and myocardial injury
- myocarditis

**Fig. 1:**
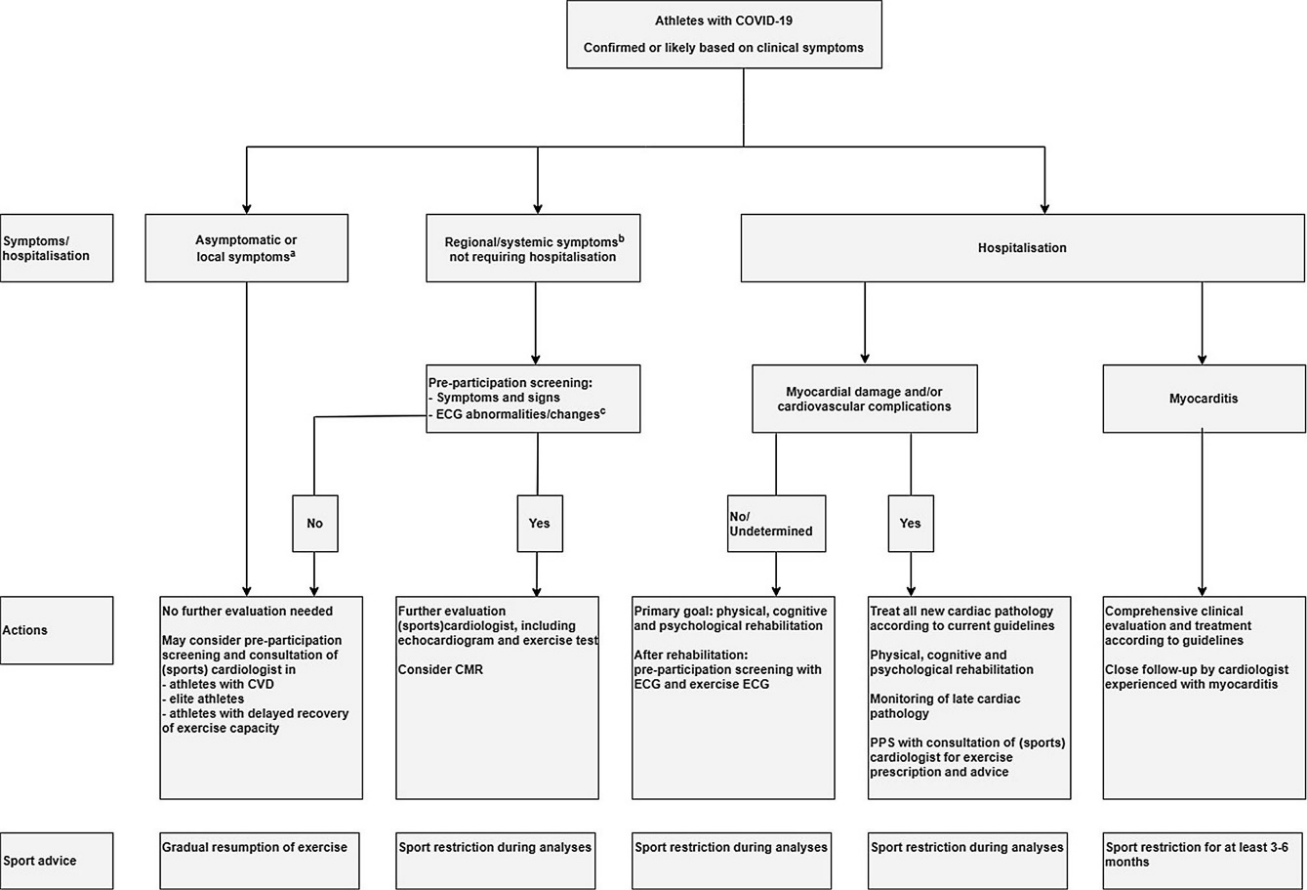
Flowchart for stratification of athletes after COVID-19 for pre-participation screening, diagnostic and therapeutic considerations. (*ECG* electrocardiogram, *CMR* cardiovascular magnetic resonance, *CVD* cardiovascular disease. ^a^Asymptomatic/non-systemic symptoms verified by critical evaluation of signs and symptoms by general practitioner or other qualified healthcare professional. ^b^See Tab. 2 for an outline of local, regional and systemic symptoms. ^c^Red flags suggestive of cardiac pathology in the 12-lead ECG are outlined in Tab. 3)

Fig. 1 shows a flowchart to assist clinicians in stratification, and suggests PPS, diagnostic and therapeutic strategies, as well as giving general sports advice.

### Asymptomatic or local symptoms (non-hospitalised)

PPS of athletes after asymptomatic infection or local symptoms (Tab. 2) of COVID-19 is not indicated if a critical evaluation of signs and symptoms (by general practitioners or other qualified healthcare professionals) is negative and shows a complete recovery. The chance of cardiac sequelae is probably negligible in such individuals. Feasibility, cost considerations and burdening of local health care systems should be considered if extending the indication for PPS to low-risk individuals. However, a PPS and consultation by a (sports) cardiologist may be considered for specific groups. These groups include, but are not limited to, athletes with pre-existent cardiovascular pathology, elite athletes and athletes with impaired recovery of exercise capacity.

**Table 2:** Symptoms

Local (nose/throat)	Regional (chest/head/neck)	Systemic
Sore throat	Dry cough	Pyrexia
Hoarseness	Wet cough (sputum/mucus)	Chills
Blocked/plugged nose	Difficulty in breathing	Anosmia/ageusia
Runny nose	Rapid breathing/shortness of breath	Myalgia/arthralgia
Sinus pressure	Chest pain	Skin manifestations (erythema, urticaria)
Sneezing	Headache	Gastrointestinal (nausea, vomiting)
Altered/loss of smell	Conjunctivitis	Encephalopathy
Altered/loss of taste		

### Regional or systemic symptoms (non-hospitalised)

PPS of patients after COVID-19 with regional or systemic symptoms (see Tab. 2 for an outline of regional and/or systemic symptoms) not requiring hospitalisation should be strongly considered. PPS includes critical evaluation of symptoms, physical examination and a 12-lead electrocardiogram (ECG). Red flags suggestive of cardiac pathology in the 12-lead ECG are outlined in Tab. 3. We emphasise that a 12-lead ECG is not the gold standard for the detection of myocarditis and other cardiovascular complication, and that a normal ECG does not rule out myocarditis in the presence of signs and symptoms suggestive of myocarditis [16, 20]. If needed, a (sport) cardiologist with experience in reading athletes’ ECGs should be consulted when differentiating between ECG changes due to cardiac adaptation to sports and exercise and ECG abnormalities suggestive of cardiac pathology [21]. Using cardiac biomarkers to screen for myocarditis has been suggested [1]. However, we advise caution when using such a screening strategy. First, most athletes do not have previously documented baseline measurements and, second, elevated biomarker levels have been demonstrated after exercise in various athletes across different sports, without clear-cut clinical implications [22].

**Table 3:** Red flags in ECG suggestive of cardiovascular complications

Tachycardia at rest
Supraventricular or ventricular arrhythmias
Ventricular ectopy
High-grade atrioventricular blocks
Multiple-lead ST elevations
T‑wave inversions
Pathological Q waves
Left bundle branch blocks
Low QRS voltage (suggestive of pericardial effusion or myocardial oedema)
Signs of right ventricular pressure overload

If the patient has completely recovered and is asymptomatic, and the 12-lead ECG is normal, gradual resumption of sports seems warranted. In the case of complaints or ECG abnormalities suggestive of cardiovascular complications, patients should be referred to a (sports) cardiologist for further evaluation. This evaluation should include at least an exercise test and an echocardiogram, but further diagnostic tests such as Holter monitoring or cardiac magnetic resonance imaging (CMR) can be considered. In the case of persistent cardiopulmonary complaints without a cardiac substrate, ruling out a pulmonary embolism should be considered.

### Hospitalised and no myocardial injury

Patients with severe COVID-19 requiring hospital or intensive care admission, without signs of myocardial damage or cardiovascular complications, should be advised to first complete a comprehensive, multidisciplinary rehabilitation programme before resuming sports and exercise [23]. After completing rehabilitation, PPS and an exercise test should be performed before the patient resumes sporting activities.

### Hospitalised and myocardial injury

In the case of myocardial damage and/or newly diagnosed cardiovascular complications during hospital admission, the primary focus should be to treat the relevant pathology according to current cardiovascular guidelines [20, 24–26]. After discharge, comprehensive rehabilitation should be prioritised with monitoring for late cardiac complications. A return to sports should take place only after a complete cardiovascular evaluation.

### Myocarditis

If SARS-CoV‑2 myocarditis is diagnosed, comprehensive clinical evaluation should take place, including CMR. In patients with myocarditis we advise intensive monitoring after discharge, and a sports restriction for at least 3–6 months, based on general myocarditis recommendations [20]. A return to sports should be evaluated by a multidisciplinary, expert team, and include input from sports cardiology and sports medicine.

## Limitations

Due to the lack of data on athletes with COVID-19, all recommendations in this point-of-view article are based on expert opinion and expert consensus. With the explosive growth of publications on COVID-19, this statement should be interpreted only in the context of the most recent peer-reviewed publications and (inter)national society recommendations. A registry of COVID-19 cases among athletes and highly active individuals, including follow-up, is needed to provide an evidence-based approach for a return to sports after recovery from SARS-CoV‑2 infection.
